# Pennsylvania’s Contribution to the Study of Immunity to Tuberculosis

**Published:** 1902-11

**Authors:** 


### EDITORIALS.

### PENNSYLVANIA'S CONTRIBUTION TO THE STUDY OF IMMUNITY TO TUBERCULOSIS.

In this issue we publish a paper by Pearson and Gilliland, on the "Immunization of Cattle against Tuberculosis," that is notable because it describes the first American work that has been done in this field. This paper accredits to de Schweinitz the honor of having first shown that animals might be rendered more resistant to tuberculosis by inoculating them with cultures of attenuated tubercle bacilli. This work was done in 1894 upon guinea-pigs, but was not pushed to a practical application. It appears that McFadyean was the first investigator to observe that cattle could be made more resistant to tuberculosis by a process of inoculation with tubercle virus, but his work was not systematized and led to no demonstration of the practical use of the process, for all of his experimental animals died of tuberculosis. Following this, von Behring, of Marburg, who had for several years been testing various drugs and toxins and suggested remedies for the cure of tuberculosis, hit upon the fact that some animals inoculated with tubercle cultures or tuberculous materials insufficient in virulence or quantity to produce fatal or even extensive disease, appeared thereafter to show heightened resistance to tubercular infection. But von Behring's experiments in this line did not begin until July, 1901, so far as one can learn from his published reports, and none of his experiments is yet terminated, as the animals are still living, and no one knows whether they are tubercular or not. The only animal of this series that von Behring has examined post-mortem was not free from tuberculosis.

The work in this direct line of Pearson and Gilliland began in September, 1900, and their experiments are the *first successful* tuberculosis immunization experiments to be completed and recorded. They have shown by a most striking experiment, fully completed and confirmed by post-mortem examination, that they have discovered a method whereby young cattle may be made highly immune to tuberculosis. The Pennsylvania State Live-stock Sanitary Board has authorized other experiments of a similar nature, and these are now being carried on for the purpose of perfecting and, if possible, of simplifying the technique of vaccination.

We congratulate the veterinary profession of Pennsylvania upon the fact that this epoch-making work has been done by two of their number, and we congratulate the stockmen of Pennsylvania upon the fact that the State is so fortunate as to have a Live-stock Sanitary Board so progressive as to make such investigations possible.

